# Efficacy and mechanisms underlying a gamified attention bias modification training in anxious youth: protocol for a randomized controlled trial

**DOI:** 10.1186/s12888-019-2224-2

**Published:** 2019-08-07

**Authors:** Julia O. Linke, Emily Jones, David Pagliaccio, Caroline Swetlitz, Krystal M. Lewis, Wendy K. Silverman, Yair Bar-Haim, Daniel S. Pine, Melissa A. Brotman

**Affiliations:** 1 Emotion and Development Branch, National Institute of Mental Health, National Institutes of Health, 9000 Rockville Pike, MSC-2670, Building 15K, Bethesda, MD 20892-2670 USA; 2 Division of Child and Adolescent Psychiatry, New York State Psychiatric Institute, Columbia University, New York, NY USA; 3 Yale Child Study Center, Yale University, New Haven, CT USA; 4 School of Psychological Sciences, Tel Aviv University, Tel Aviv, Israel

**Keywords:** Attention bias modification, Cognitive behavioral therapy, Randomized controlled trial, Anxiety, Youth, fMRI, Gamification

## Abstract

**Background:**

Attention bias modification training (ABMT) and cognitive behavioral therapy (CBT) likely target different aspects of aberrant threat responses in anxiety disorders and may be combined to maximize therapeutic benefit. However, studies investigating the effect of ABMT in the context of CBT have yielded mixed results. Here, we propose an enhanced ABMT to target the attentional bias towards threat, in addition to classic CBT for anxiety disorders in youth. This enhanced ABMT integrates the modified dot-probe task used in previous studies, where a target is always presented at the previous location of the neutral and not the simultaneously presented threatening stimulus, with a visual search, where the targets are always presented distally of threatening distractors. These two training elements (modified dot-probe and visual search) are embedded in an engaging game to foster motivation and adherence. Our goal is to determine the efficacy of the enhanced ABMT in the context of CBT. Further, we aim to replicate two previous findings: (a) aberrant amygdala connectivity being the neurobiological correlate of the attentional bias towards threat at baseline; and (b) amygdala connectivity being a mediator of the ABMT effect. We will also explore moderators of treatment response (age, sex, depressive symptoms and irritability) on a behavioral and neuronal level.

**Methods:**

One hundred and twenty youth (8–17 years old) with a primary anxiety disorder diagnosis all receive CBT and are randomized to nine weeks of either active or control ABMT and symptom improvement will be compared between the two study arms. We will also recruit 60 healthy comparison youth, who along with eligible anxious youth, will be assessed with the dot-probe task during fMRI (anxious youth: before and after training; healthy volunteers: second measurement twelve weeks after initial assessment).

**Discussion:**

The present study will contribute to the literature by (1) potentially replicating that aberrant amygdala connectivity mediates the attentional bias towards threat in anxious youth; (2) determining the efficacy of enhanced ABMT; and (3) advancing our understanding of the mechanisms underlying ABMT.

**Trial registration:**

Clinicaltrials.gov: NCT03283930 Trial registration date: September 14th 2017. The trial registration took place retrospectively. Data acquisition started February 1st 2017.

## Background

Anxiety disorders, such as separation anxiety, specific phobia, social phobia, generalized anxiety and panic, occur in 15–20% of all children and adolescents [1]. This is particularly important because anxiety during childhood and adolescence predicts not only later anxiety, but also other psychiatric disorders during adulthood [2, 3]. Cognitive behavioral therapy (CBT) emphasizes behavioral exposures and cognitive restructuring that aim to change dysfunctional behaviors (e.g. avoidance of fear-provoking stimuli/situations) and thoughts (e.g., catastrophic thinking). CBT has been shown to produce medium-to-large symptom reductions in anxious children and adolescents [4]. However, only one in five clinically anxious youth receives treatment [5] and about 50% of treated patients do not respond to treatment [6, 7]. This has motivated researchers to develop Attention Bias Modification Trainings (ABMT), which specifically target one well-replicated mechanism in anxiety disorders: an attentional bias towards threat [8].

ABMT is typically employed using a modified dot-probe task [9]. The dot-probe task indexes attentional bias using the difference in reaction times to target stimuli presented at the previous location of either a threatening or neutral stimulus, often emotional faces [10], for details see Fig. 2b). During ABMT, the probe consistently appears in the location of the neutral face stimulus thereby implicitly training participants to attend away from the threatening face, whereas in the control condition the probe replaces the neutral and threatening face stimuli with equal probability [11]. Thus, the modified version of the dot-probe task is hypothesized to change the attentional bias ‘bottom up’ by directly retraining salience contingencies [11]. On a neurobiological level, the dot-probe task has been most robustly associated with amygdala-insula connectivity [12–14], which according to a first report by White and colleagues does not only differentiate anxious from healthy youth, but also predicts the response to ABMT [15]. Of note, the visual search for non-threat targets in the context of threatening distractors has also successfully been used as ABMT [16, 17], but has been more related to activity in the fronto-parietal attention network and the amygdala [18, 19]. Given that the modified dot-probe task and the visual search training target different, albeit partial overlapping neural circuits, it is conceivable that a combination of both trainings would yield enhanced treatment effects.

To date, seven meta-analyses have investigated the effects of ABMT in terms of the modified dot-probe task in youth and adults showing small [20–23] to moderate [24–26] reductions in attention bias towards threat. ABMT effects on symptoms of anxiety were generally small [20–26], albeit single reports of moderate [27], and no symptom reductions [28]. An additional six studies have investigated the efficacy of active vs. control ABMT in addition to CBT. Four studies including the one by White and colleagues (2017) showed an enhanced effect of the combination treatment on symptoms rated by clinicians [15, 29–31]. An additional study showed improved effects of ABMT+CBT on symptoms rated by child and parent only [32]; one study reported no significant differences between active ABMT+CBT vs. control ABMT+CBT [33].

These mixed findings might be partially explained by differences in the study designs. Particularly, the vertical, rather than horizontal, presentation of stimulus pairs during the modified dot-probe task appears to yield larger effects [27]. Moreover, effect sizes of ABMT are larger in laboratory vs. home settings, and when anxiety symptoms are assessed by clinicians (rather than self-report) [34]. Inconsistencies may further relate to individual differences in task engagement and motivation to complete a specific task [35], as engagement has been shown to moderate the efficacy of cognitive trainings [36] such as the modified dot-probe task [37]. The use of game-like elements, henceforth referred to as “gamification”, [38] could make ABMT more engaging and motivating, thereby enhancing adherence to the training and improving ABMT efficacy [39]. As outlined by Boendermaker and colleagues (2015), the use of game elements in cognitive trainings such as ABMT should ideally target both extrinsic and intrinsic motivation, which might be achieved by using a performance-based point system and designing the training in a way that it is inherently interesting or enjoyable. The use of game-elements seems particularly promising in younger children (e.g. < 12 years), who show relatively small effects compared to older youth [40].

Finally, it has been argued that ABMT can only result in anxiety symptom reduction, when an attentional bias towards threat is initially present, i.e. the proposed target mechanism of ABMT [41]. Unfortunately, it is difficult to assess attentional bias behaviorally as reliability of the bias measure derived from the dot-probe task is poor [42]. However, reliability of amygdala connectivity as a neurobiological correlate of the attention bias is better [43].

### Aims

There are three aims of this study. First, [1] we aim to test the efficacy of an enhanced ABMT, which consists of a gamified combination of the modified dot-probe task and a visual search, in a clinic setting in anxious youth. We hypothesize that children receiving active ABMT+CBT will show greater improvement relative to those receiving control ABMT+CBT, reflected by greater decreases in the Pediatric Anxiety Rating Scale (PARS [44]) scores and the Clinical Global Impressions-Improvement Scale (CGI-I [45]) scores rated by clinicians. Second, we seek to replicate the results previously reported by White and colleagues (2017): (2a) aberrant amygdala connectivity being the neurobiological correlate of the attentional bias towards threat at baseline; and (2b) amygdala connectivity being a mediator of the ABMT effect. To establish the association between anxiety and aberrant amygdala connectivity related to the attentional bias towards threat at baseline (aim 2a), we will also recruit a healthy comparison sample. Third, [3] we will explore moderators of treatment response namely age, sex, depressive symptoms, and irritability on a behavioral and neural level.

## Methods

### Study setting and trial design

**Fig. 1:**
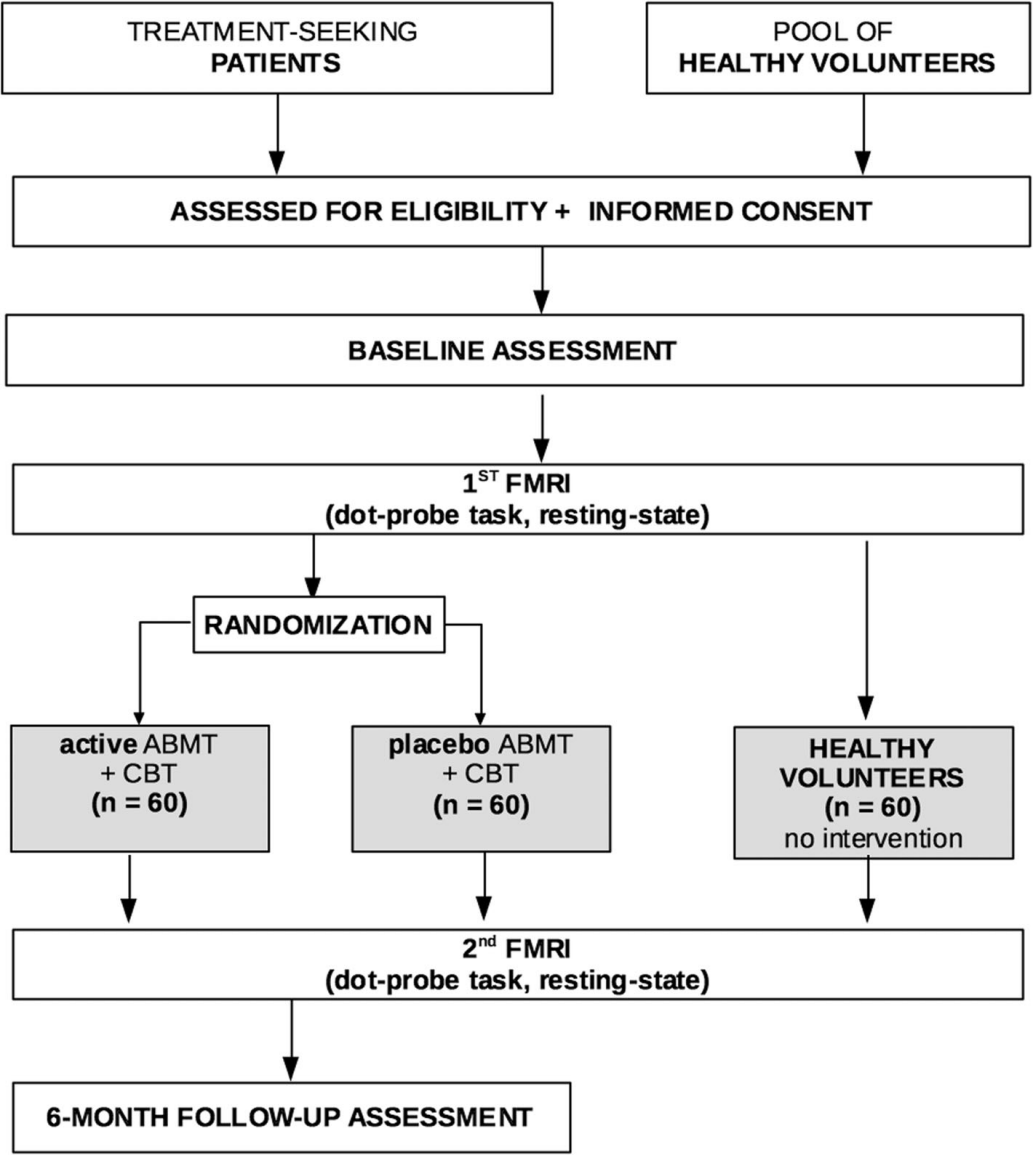
Study flow chart

This randomized controlled trial is being conducted at the National Institutes of Mental Health in Bethesda, Maryland, United States of America. The study adheres to the Consolidated Standards of Reporting Trials (CONSORT) statement [46] and the SPIRIT guidelines (Standard Protocol Items: Recommendations for Interventional Trials) [47]. The study protocol, information on the study, informed consent, and trial-related documents were approved by the Institutional Review Board of the National Institute of Mental Health. We chose a design similar to White and colleagues (2017); random assignment of anxious youth to one of two treatment-arms (active ABMT + CBT vs. control ABMT + CBT); assessment of anxiety and symptom severity pre-, mid-, and post treatment and 6 month after treatment; assessment of functional magnetic resonance imaging (fMRI) data pre- and post-treatment; recruitment of a healthy sample to test the association between anxiety and amygdala connectivity at baseline. A flowchart of the study is shown in Fig. 1.

### Sample size

To test the first hypothesis regarding the efficacy of the enhanced ABMT, the target sample size was estimated using G*Power version 3.1 [48] to achieve a between-group (active vs. placebo ABMT) effect size of Cohen’s d = 0.45 (as reported by [15]), with a power of 80%, and an alpha error rate of .05. This calculation generated a sample size of *N* = 120. We will analyze the data with a linear mixed model that allows for both the fixed treatment effect and random subject effects on anxiety levels.

We also calculated the sample size needed to test the effect of the active vs. control ABMT within a linear mixed model using the PARS baseline ratings as covariate with the GLIMMPSE software [49]. For this secondary analysis, we used the following parameters: α = .05, β = .80, equal group size, and information about correlations between the PARS scores obtained from White et al. 2017 (PARS week 0 with PARS week 4: r = .46, PARS week 0 with PARS week 8: r = .20, PARS week 4 with PARS week 8: r = .48). As outlined above, we expect a larger treatment effect size and/or reduce variability in the treatment effect compared with White and colleagues (2017), as we provide an enhanced ABMT that consists of the modified dot-probe and a visual search task and is, further, enriched by game elements to increase engagement and adherence. These analyses showed that the effect might already be detected with *N* = 86 (effect size * 1.25, variability * 0.75) or *N* = 112 (effect size * 1.25, same variability). Depending on the speed of the participant accrual and the external review of our group in 2021, it might be necessary to have an independent assessor conduct an interim analysis, either when N = 86 or when *N* = 112. However, we will remain blind to our data, and the final analysis will be completed upon attaining the proposed sample size of *N* = 120.

To test hypothesis 2a that aberrant amygdala connectivity is the neurobiological correlate of the attention bias in anxious youth [15], we will also acquire anxiety ratings and functional magnetic resonance imaging (fMRI) data during the dot-probe task from healthy volunteers. White and coworkers (2017) were able to show this effect comparing 54 anxious to 51 healthy youth. Thus, we aim to acquire imaging data from 60 healthy volunteers (HV) to replicate this finding.

### Recruitment and eligibility criteria

#### Recruitment and eligibility

Recruitment for this study began in February 2017. The study is being advertised in a local parenting magazine, through talks at local schools and flyers. After requesting information, interested families receive information about the trial and are asked to provide general information regarding the potential study participant, such as age and presenting anxiety symptoms. If eligibility criteria (Table 1) are met based on this initial assessment, families are invited onsite.

**Table 1:** Eligibility criteria

Inclusion criteria	Exclusion criteria
• Age: 8–17 (participants, who consent as 17-year-olds but turn 18 during the course of the study, will be eligible to complete all procedures) • IQ: all participants will have IQ-scores > 70 as assessed by the WASI • Language: all participants will be fluent in English • Any current anxiety diagnosis for patient group only	• Any serious medical condition or condition that interferes with fMRI scanning • Pregnancy • Current use of any psychoactive substance • Current suicidal ideation • Current diagnosis of ADHD of sufficient severity to require pharmacotherapy, Tourette’s Disorder, OCD, PTSD, conduct disorder, major depressive disorder • Past or current history of mania, psychosis, or severe pervasive developmental disorder

Abbreviations: *ADHD* attention-deficit hyperactivity disorder, *IQ* intelligence quotient; *OCD* obsessive compulsive disorder, *PTSD* post-traumatic stress disorder, *WASI* Wechsler Abbreviated Scale of Intelligence

During an onsite visit, participants and their parent(s) are administered modules of the Anxiety Disorders Interview Schedule – Child and Parent (ADIS-C/P) [50] to assess anxiety disorders and the Kiddie Schedule for Affective Disorders and Schizophrenia (K-SADS) [51] (without the anxiety modules) to determine current or past comorbidities. Assessment interviews are conducted with parents and children independently by a trained clinician. Diagnoses are then confirmed by D.S.P.. Further, trained research assistants administer the Wechsler Abbreviated Scale of Intelligence [52]. Once eligibility is confirmed, clinicians discuss treatment options and assess whether patients are suitable for CBT (e.g. willing to compete exposures) and ABMT (physically and behaviorally able to complete ABMT reliably). Then, parents give written informed consent and the children provide written assent.

Next, participants undergo three psychoeducational sessions and the subset who does not have MRI contraindications is assessed with the dot-probe task during fMRI. Prior to randomization and the beginning of active treatment, clinicians determine the participant’s willingness to engage in the intervention and ability to regularly participate in treatment sessions. If patients are unwilling or unable to participate in the treatment trial, alternative treatment options are offered (i.e., psychotropic medication, CBT without ABMT, community referrals).

All inclusion and exclusion criteria that apply to the patients also apply to the healthy volunteers (HV) and are assessed in the same way. However, HV must not fulfill criteria for a current or lifetime psychiatric disorder, which is confirmed by a trained clinician administering the ADIS and KSADS to the child and the parent. Further, MRI contraindication is exclusionary for HV.

### Randomization and blinding

After baseline screening and three psychoeducational visits, patients are randomized to either the active or control ABMT condition. Randomization is handled by a computer algorithm administered by a person independent of the researchers and treating clinicians. Thus, patients, experimenters, and clinicians are blind to the AMBT condition (active vs. control) that participants receive. In case of an interim analysis, the blind will only be lifted for the person conducting the analysis, who will not be in contact with the patients.

### Measures

#### Primary outcome measures

The Clinical Global Impression of Improvement Scale (CGI-I) is a measure of global symptom improvement rated by clinicians [45]. Its score ranges from 1 to 7, with lower scores reflecting greater levels of improvement. This scale provides an ordinal outcome as participants with CGI-I ratings of ≤3 at week 8 are considered as “responder” and participants with scores > 3 are counted as “non-responder”.

The Pediatric Anxiety Rating Scale (PARS) measures anxiety symptoms and related functional impairment in youth as continuous outcome [44]. It comprises a 50-item checklist asking for seven dimensions of global severity/ impairment: 1) number of symptoms, 2) frequency of symptoms, 3) severity of distress associated with anxiety symptoms, 4) severity of physical symptoms, 5) avoidance, 6) interference at home, and 7) interference outside of home. Each item is rated on a 0–5 scale by a clinician based on parent- and child-report. The sum score is calculated based on sub-scales 2, 3, 5, 6, and 7 and ranges from 0 to 25 with higher scores reflecting greater levels of anxiety. Scores above 11.5 indicate the presence of an anxiety disorder. The PARS shows satisfactory internal consistency (intraclass correlation coefficient [ICC] = 0.97) and moderate test-retest reliability (.55) [44]. If findings for these two primary outcome measures are discrepant, more weight will be given to the PARS, given the greater statistical power with the continuous approach.

#### Secondary outcome measures

The Children’s Global Assessment Scale (CGAS; [53]) is a clinician-rated measure of global functioning, which scores range from 1 to 100 and shows good interrater reliability (ICC = 0.88) [54]. Higher CGAS-scores reflect better overall functioning [55].

The Screen for Child Anxiety Related Disorders (SCARED; [56]) is a 41-item, dual-informant measure of pediatric anxiety symptoms; we use the mean of the parent and child ratings. The questionnaire consists of five subscales assessing symptoms of generalized anxiety, social anxiety, separation anxiety, panic, and school refusal. Items are rated on a scale from 0 to 2; the overall score range is 0–82. Higher scores reflect greater levels of anxiety, and scores above 25 indicate the presence of an anxiety disorder. The SCARED has good internal consistency (α = .91) [57] and good to excellent test-retest reliability (.70–.90) [58].

The State-Trait Anxiety Inventory for Children (STAI-C; [59]) is a 20-item self-report measure of trait anxiety. Items are rated on a scale from 1 to 3; the overall score range is 20–60. Higher scores reflect greater levels of anxiety. The STAI-C has good internal consistency (α = .86) and test-retest reliability estimates range from poor to good (.31–.86) [60].

The Self-Efficacy Questionnaire (SEQ-C; [61]) is a 24-item self-report measure of self-efficacy in youth. The questionnaire is made up of three subscales assessing social self-efficacy, academic self-efficacy, and emotional self-efficacy. Items are rated on a scale from 1 to 5; the overall score range is 24–130. Higher scores reflect higher levels of self-efficacy. The SEQ-C has good internal consistency (Cronbach’s α = .88) [61].

#### Measures of comorbid symptom clusters

Elevated anxiety levels frequently co-occur with increases in depressive symptoms [62] and increased irritability [63]. We will determine whether symptoms of depression assessed with the Mood and Feelings Questionnaire (MFQ; [64]) and irritability measured with the Affective Reactivity Index (ARI; [65]) at baseline moderate training effects. Further, we will investigate whether training effects transfer to these symptom clusters mid- and post-treatment.

The MFQ is a 33-item dual-informant measure of depressive symptoms in children. Items are rated on a scale from 0 to 2; the overall score range is 0–66. Higher scores reflect higher levels of depression, and scores above 27 indicate clinically significant depression. The MFQ has excellent internal consistency (α = .91 to .93) [66].

The ARI is a 7-item dual-informant measure of irritability in children. Items are rated on a scale from 0 to 2; the overall score range is 0–12. Higher scores reflect higher levels of irritability. The ARI has good to excellent internal consistency (α = .88–.92) and test-retest reliability (.88–.90) [67].

### Treatment

#### Cognitive behavioral therapy

All participants receive 12 weekly sessions of CBT [68] administered by one of the licensed psychologists who specialize in the treatment of anxiety in youth (one of them being K.L.). Each CBT session is 40 to 60 min and the youth are assigned additional home practice, which builds on the exposure completed during the sessions. Treatment begins with an introduction to CBT, psychoeducation, and self-monitoring. After three weeks of treatment, children are instructed to complete their first out-of-session exposure. In-session exposures and cognitive restructuring exercises begin at session four. Table 2 gives an overview of the treatment, highlighting the skills and exercises emphasized in each session. Parents were involved in three sessions across treatment, which involved assessment of child psychopathology and improvement across the treatment. Home practice information was relayed to parents so that they could remind the kids to complete their exposures; however, the treatment was primarily individually focused. The CBT therapists were trained on the CBT protocol during a one-day training at the Child Study Center at Yale by W.K.S., who will also provide supervision as needed. There will be a bi-weekly intervision, where D.S.P., K.M.L., and another treating clinician will discuss the treatment progress and determine the need for supervision.

**Table 2:**
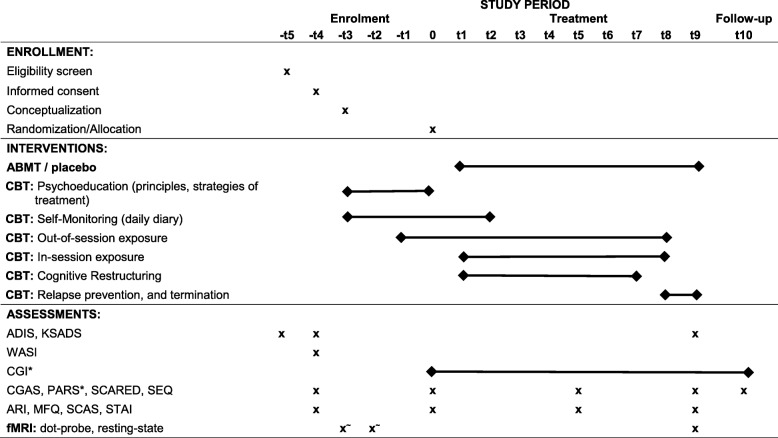
Schedule of enrolment, interventions, and assessments

Abbreviations: *ADIS* Anxiety Disorders Interview Schedule, *ARI* Affective Reactivity Index, *CGAS* Children’s Global Assessment Scale, *CGI-I/S* Clinical Global Impressions-Improvement/Severity, *K-SADS* Kiddie-Schedule for Affective Disorders and Schizophrenia-Present and Lifetime Version, *MFQ* Mood and Feelings Questionnaire, *PARS* Pediatric Anxiety Rating Scale, *SCARED* Screen for Child Anxiety Related Disorders, *SCAS* Spence Child Anxiety Scale, *SEQ* Self-Efficacy Questionnaire, *STAI-Trait* State-Trait Anxiety Inventory Trait version, * indicates the main outcome measures

#### Attention bias modification training

**Fig. 2:**
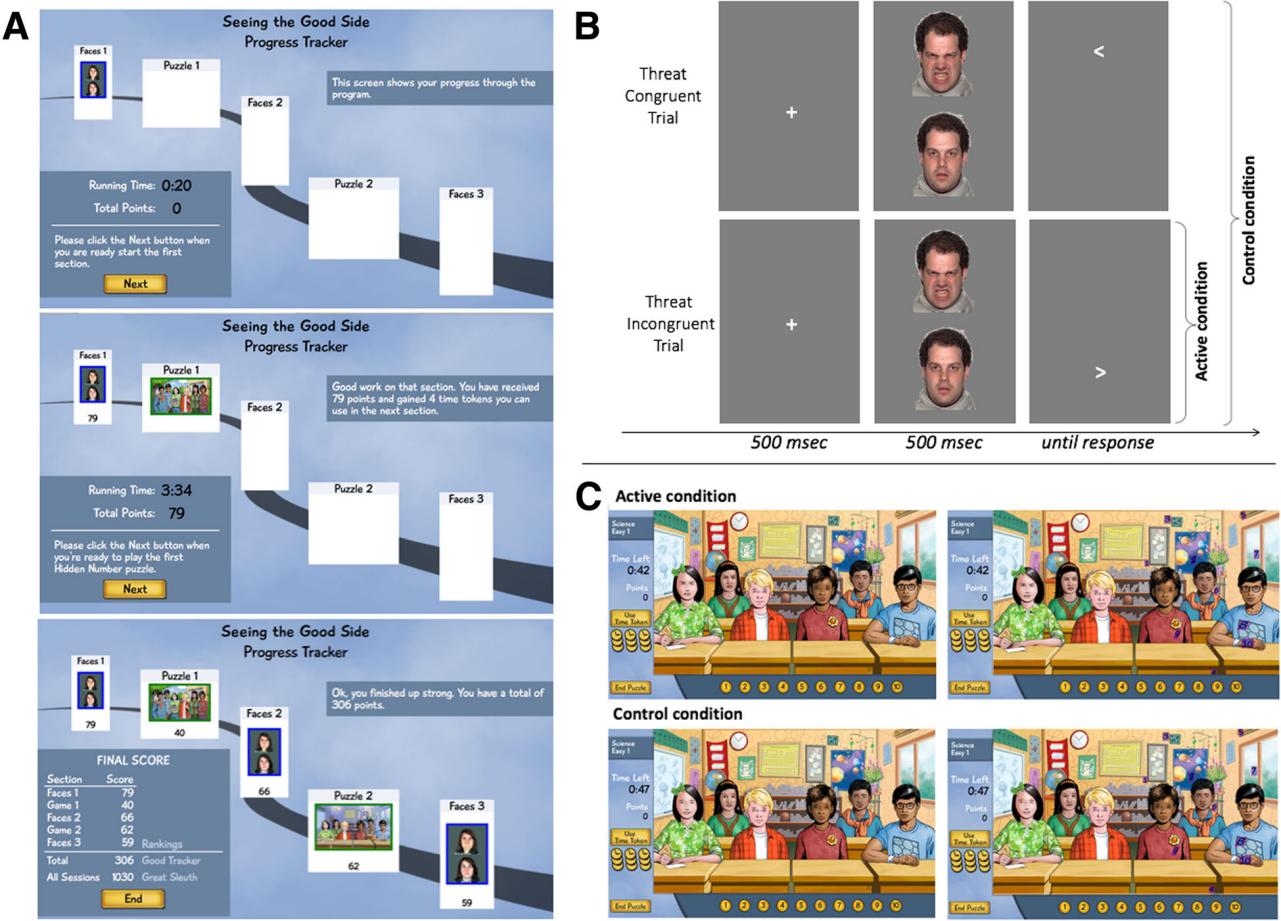
Depiction of the gamified ABMT. **a** Shows the general outline of the training and feedback provided as participant progress through the training. **b** Shows the modified dot-probe task. The active condition consists of threat incongruent trials and neutral-neutral trials only, whereas the congruent and incongruent trials are shown with equal probability during the control condition. The face stimulus stems from the NimStim stimulus set [72]. **c** The upper panel shows the active visual search with two threatening faces and the lower panel shows the control condition with neutral faces only. At the left you see the same view as the participants and at the right searchable numbers are highlighted

As noted above, patients are randomly assigned to receive active or control ABMT that is administered via laptop prior to every CBT session (Table 2). The ABMT is administered to the youth by a research assistant (blinded to ABMT condition) who is responsible for setting up the computer. During task completion the research assistant remains in the test room but cannot see the screen in order to remain blinded. Both active and control ABMT are embedded within a game that consists of A) three 80-trial blocks of the modified dot-probe task [10], and B) two sessions of the visual search game (Fig. 2) in ABABA order. Each training session lasts 10–16 min.

During each of the three dot-probe blocks, participants are presented with pairs of faces (60 angry-neutral and 20 neutral-neutral) from picture-set A of the TAU-NIMH Attention Bias Measurement toolbox [10] are shown vertically on a computer screen (1440 × 900 pixel) for 500 msec. Next, a probe (< or >) will appear in the location vacated by one of these faces. Participants are required to indicate the direction the arrow probes are pointing to as quickly and as accurately possible. The response is followed by a fixation cross presented for 500 msec. In the control condition, trial type (angry-neutral, neutral-neutral), angry-face location (top or lower part of the screen), and probe location (behind an angry or neutral face at the top or lower part of the screen) are fully counterbalanced. In the active condition, the target appears at the neutral-face location in all angry-neutral trials, thereby training the participant to direct attention away from the angry face.

As it the primary aim of this study to replicate the efficacy of modified dot-probe task for the treatment of anxiety in youth, the actual training remains unchanged but is augmented by a performance-based point system. In each of the three blocks, participants can earn up to 80 points if they respond correctly to the probe. In addition, participants can earn “time tokens” for responding correctly and quickly in the modified dot-probe task. The number of time tokens are determined by (a) the number of correct responses, which is multiplied with 0.04, (b) the reaction time (number of time tokens = (600 msec – response time in msec)/ 50), and (c) the number of errors; 10 tokens are subtracted for each error after the first 4 errors. These tokens may be used in the visual search to extend the search time limit by 10 s each by clicking on a clock icon on the side panel. Points that are earned during the last dot-probe block do not translate into time points but count towards the final score of the session and the score across all sessions (see Fig. 2a).

As outlined above, our novel ABMT combines the modified dot-probe task with a visual search. Of note, the visual search task has been designed as a puzzle with a level-structure comparable to actual games people play to be inherently interesting. We expect that participants will be intrinsically motivated for this part of the “game” and see the dot-probe part as a mean to spend more time on the visual search thereby increasing the extrinsic motivation to perform well on the dot-probe too.

Within the visual search task, participants must find all numbers hidden within a scene in a limited time (level 1: 1 min for 10 numbers; level 2: 1 min 15 s for 15 numbers; level 3: 1 min 30 s for 20 numbers). The visual search scenes depict 6 youth with different ethnicities engaged in school-related activities. In the active condition, four youth display neutral and two display angry facial expressions and numbers can be found distal to the angry and proximal to the neutral faces. In the control condition all faces have a neutral expression and numbers can be found at the same location as in the active condition. For each number found, participants earn additional points (level 1: 10 points, level 2: 15 points, level 3: 20 points). For clicking on something other than the hidden numbers points are deducted (level 1: 1 point, level 2: 2 points, level 3: 3 points). Moreover, they can earn a bonus of 10 points for finding all numbers before the time is up. In between the dot-probe and visual search blocks feedback regarding points and time tokens is provided. At the end of each training session, participants receive feedback regarding their total score on that day and their overall score across all training sessions. The overall score (points from the dot-probe and the visual search task) does not translate into tangible rewards as there is evidence that these might undermine intrinsic motivation for a task [69].

It is possible that some participants infer which condition they have been allocated to from the matching of the probes to the stimuli. Therefore, we ask them before the debriefing to guess whether they think they had been allocated to the active or the placebo condition and how confident they are that their guess is correct. This information might be used in additional exploratory analyses.

### Functional magnetic resonance imaging

#### Attention Bias assessment – dot-probe task

Participants complete an event-related dot-probe fMRI task [10] at baseline and after treatment. This dot-probe task is precisely the same as the one we use during the training, except that here we use picture set B of the TAU-NIMH Attention Bias Measurement toolbox [10], present the task in 2 blocks/ runs of 120 trials, and use a longer inter-trial interval with an average of 2 s to better capture the BOLD response. The task has three conditions that are presented in random order: 1) 40 congruent trials, when the arrow probe appears in the location of the angry face; 2) 40 incongruent trials, when the arrow appears in the location of the neutral face; and 3) 40 neutral trials, with two neutral faces as a non-threat condition. We are particularly interested in contrasting incongruent vs. congruent trials, as a measure of “attention bias” that reflects differential brain function when attention is allocated away or toward threat (angry face). Each task condition consists of 80 trials that are presented across two runs. Data will be excluded from participants who respond incorrectly on over 25% of trials.

#### Data acquisition

Functional neuroimaging data are acquired with a 3 T GE scanner (Waukesha, Wisc.) with an eight-channel head coil with 2.5 mm resolution and T2* weighting (TR = 2300 ms, TE = 25 ms, flip angle = 50°, FOV = 240 mm^2^, matrix = 96 × 96, 41 contiguous 3-mm interleaved axial slices). For co-registration and normalization, we acquire a a high-resolution T1-weighted 3D standard sequence (slice thickness 1 mm, FOV = 256 × 256 × 176 mm, matrix = 256x256x176).

#### Preprocessing of task-based fMRI data

Task-related functional imaging data are preprocessed with the AFNI (Analysis of Functional Neuroimages) software. Steps include slice timing correction, nonlinear registration of echoplanar data to anatomical scans, normalization, and spatial smoothing. Individual-level statistical analyses are carried out using a general linear model, with regressors for correct trials for each task conditions (congruent, incongruent, neutral) and one regressor for incorrect trials. Further, the models include regressors accounting for baseline drift and head motion. We also separately assess task-based functional connectivity of the left and right amygdala for the congruent, incongruent, and neutral conditions using generalized psychophysiological interaction (gPPI) analysis [70].

### Statistical analyses

#### Behavioral data: efficacy of the ABMT

We will perform both per protocol analyses (i.e., evaluating only participants who completed the study) and intent to treat analyses (i.e., evaluating all participants enrolled regardless of whether they complete the study). PARS rating data will be entered into a linear mixed model with ABMT group (active, placebo) as a between-group factor and time as within-subject variable. If there are significant differences in pretreatment ratings, they will be included as a covariate and time will be a two-level factor (mid- and post-treatment). If there are no differences in pretreatment ratings, time will be entered as a three-level factor (pre-, mid- and post-treatment). The efficacy of the training will be determined based on the contrast that tests for group differences in the posttreatment PARS ratings. Additionally, we will compare responders (CGI-I score ≤ 3) and non-responders (CGI-I score > 3) between active and control group using a Chi-square test. Additional analyses will explore effects of age and sex on treatment response.

#### Imaging data

Consistent with previous studies [12–15] showing aberrant amygdala connectivity in anxiety and greater stability of amygdala-based connectivity compared to activation on the dot-probe task [43], our analysis will focus on task-related amygdala-seed functional connectivity. We are particularly interested in how amygdala connectivity at baseline 1) differs between patients and healthy comparison participants, 2) predicts overall treatment response in patients, and 3) relates to ABMT-specific treatment effects. We will also compare amygdala-seed connectivity pre- vs. post-treatment. Additionally, we will explore treatment effects on amygdala activation and effects of age, sex, and other clinical measures (MFQ, ARI) on brain function and treatment-related changes in brain function.

All whole brain analyses will use a voxel-wise threshold of *p* < 0.001 and cluster correction to achieve a whole brain *p* < 0.05 false positive rate. The number of voxels in a cluster will be determined using 10,000 Monte Carlo simulations using AFNI’s 3dClustSim tool with the autocorrelation function correction. With regard to previous findings, we will use a region-of-interest approach to test for significant results specifically in the prefrontal cortex and the insula [15]. Consistent with previous studies the cluster-wise threshold for the prefrontal cortex will be based on a prefrontal cortex mask [15, 43]. Group maps will be thresholded to include only data for which > 90% of participants had valid data.

Differences in pretreatment amygdala connectivity will be tested with a linear mixed-effects model using AFNI’s 3dLME program using group (anxious patients vs. healthy comparison participants) as a between-subject variable and task condition (congruent, incongruent, neutral) as the within-subject variable. The question of whether pre-treatment amygdala connectivity predicts treatment response will be tested in the patient group using 3dMVM with post-treatment PARS ratings as a continuous variable, ABMT group (active, placebo) as a between-subject variable, and task condition (congruent, incongruent, neutral) as the within-subject variable. To control for baseline anxiety, pre-treatment PARS rating will be included as a covariate.

We will test two interactions of interest: [1] the two-way task condition-by-posttreatment PARS interaction will be examined to identify connectivity changes related to overall treatment response; and [2] the three-way task condition-by-ABMT-by-posttreatment PARS rating interaction will be examined to assess connectivity related to treatment differences that differs between the active and control ABMT groups. Post-hoc visualization will rely on correlations between connectivity levels and posttreatment PARS ratings for each of the two ABMT groups.

## Discussion

Attentional bias towards threat is a key mechanism in anxiety disorders [8] that is not specifically targeted by CBT. ABMT procedures mostly using a modified version of the dot-probe task have been specifically developed to change this attentional bias ‘bottom up’ by directly retraining salience contingencies. Despite promising results of initial studies, this has not proved straightforward [28] and thus further research is warranted.

This study will be the largest randomized controlled trial in anxious youth that uses fMRI to assess the attention bias by focusing on amygdala connectivity – a neurobiological marker that has adequate test-retest reliability [43]. Of note, the intervention used in this study has been designed to maximize treatment response and therefore consists of a novel combination of the modified dot-probe task and a modified visual search task to more comprehensively target processes involved in the selective allocation of attention. Moreover, we also address the issue of low motivation/ low adherence by designing the visual search training as a puzzle that will be inherently interesting and additionally introducing game elements such as digital rewards, feedback, levels, and time pressure. Finally, we apply several strategies shown to enhance the ABMT effect including [1] using vertical presentation of stimuli pairs during the modified dot-probe task [27]; [2] training in a clinic setting to minimize distractions [8], and activate a relevant ‘fear structure’ to provoke the attentional bias that can then be modified [30]; and [3] using of independent clinician-based assessments of anxiety, as opposed to self-reported anxiety measures that are more prone to biased reporting [71]. Considering this framework, it can be expected that the results of this study will have a significant impact on the debate regarding the efficacy of ABMT on attentional biases. The study also can contribute to the development of personalized treatments and delineate the mechanisms underlying a predictive relationship between pretreatment attentional bias reflected not only in behavioral measures, but also increased amygdala-insula connectivity and response to ABMT.

Despite considerable strengths, there are several limitations inherent in the study design. First, we essentially combine three interventions (modified dot-probe, positive visual search, and CBT), and test them with two study arms. Thus, it will not be possible to dissociate effects of the modified dot-probe aiming at bottom-up attentional processes, the visual search potentially also targeting top-down attentional processes and CBT meant to reduce dysfunctional appraisal and avoidance of potential threats using exposure and cognitive strategies such as restructuring. In this regard, we also will not be able to answer whether the gamification led to an enhanced treatment response should we find one. A second related limitation is that the attention bias will be solely assessed with the dot-probe task. No task measuring goal-directed inhibitory control of attention potentially targeted by the visual search training will be administered as the sum of the ratings, interview and the dot-probe task that are conducted pre- and post-treatment already pose such a strain on the participating families that unfortunately the assessment of a second task pre-post treatment is practically not feasible. Thirdly, all patients will receive CBT with either active or control ABMT, so our findings may not be generalizable to other effective treatments, such as medication. Fourth, we enroll participants with any one of various anxiety disorders (generalized anxiety disorder, social phobia, separation anxiety, specific phobia). For this reason, we cannot tailor training stimuli to specific anxiety disorders. Fifth, the performance during the visual search task depends on the performance during the dot-probe task. Although the dot-probe task is easy (participants earn a point for correctly indicating whether the arrow is pointing to the left or the right) and participants always earn points, which they can use during the visual search task; this might negatively impact the motivation for some participants. However, only the numbers found during the regular time will be analyzed. Sixth, it is not the focus of the present study to assess the effect of the game elements. Therefore, engagement and adherence with regards to the game elements are not specifically assessed or evaluated but might be the focus of future studies. Lastly, the generalizability of our findings may be affected by the study’s exclusionary criteria (e.g., depression, OCD).

In summary, this is an important study that further tests the efficacy of a computer-based attention bias modification training aiming at reducing increased attention towards threat – a mechanism not specifically targeted by CBT. Given previously mixed findings, we employ a combination of the modified dot-probe and visual search to target both bottom-up and top-down attentional processes. Additionally, the training will be imbedded in a game to enhance motivation and adherence. Finally, we will use fMRI to delineate mechanisms relevant for future guided treatment selection in this largest randomized controlled trial in anxious youth to date.

## Data Availability

Not applicable.

## Abbreviations

ABMT: Attention Bias Modification Training
ADIS: Anxiety Disorders Interview Schedule
AFNI: Analysis of Functional Neuroimages
ARI: Affective Reactivity Index
CBT: Cognitive behavioral therapy
CGAS: Children’s Global Assessment Scale
CGI: Clinical Global Impressions-Improvement Scale
CONSORT: Consolidated Standards of Reporting Trials
fMRI: functional magnetic resonance imaging
FoV: Field of view
FWHM: Full width at half maximum
gPPI: generalized psychophysiological interaction
HV: healthy volunteers
K-SADS: Kiddie Schedule for Affective Disorders and Schizophrenia
MFQ: Mood and Feelings Questionnaire
PARS: Pediatric Anxiety Rating Scale
RUPP: Research Unit on Pediatric Psychopharmacology
SCARED: Screen for Child Anxiety Related Disorders
SEQ-C: Self-Efficacy Questionnaire
SPIRIT: Standard Protocol Items: Recommendations for Interventional Trials
STAI-C: State-Trait Anxiety Inventory for Children
TE: Echo time;
TR: Repetition time
